# Comprehensive analysis of differentially expressed profiles of lncRNAs and construction of miR-133b mediated ceRNA network in colorectal cancer

**DOI:** 10.18632/oncotarget.15045

**Published:** 2017-02-03

**Authors:** Hao Wu, Runliu Wu, Miao Chen, Daojiang Li, Jing Dai, Yi Zhang, Kai Gao, Jun Yu, Gui Hu, Yihang Guo, Changwei Lin, Xiaorong Li

**Affiliations:** ^1^ Department of Gastrointestinal Surgery, The Third Xiangya Hospital of Central South University, Changsha, Hunan 410013, P. R. China

**Keywords:** miR-133b, lncRNA, ceRNA, colorectal cancer

## Abstract

**Background:**

Growing evidence suggests that long non-coding RNAs (lncRNAs) play a key role in tumorigenesis. However, the mechanism remains largely unknown.

**Results:**

Thousands of significantly dysregulated lncRNAs and mRNAs were identified by microarray. Furthermore, a miR-133b-meditated lncRNA-mRNA ceRNA network was revealed, a subset of which was validated in 14 paired CRC patient tumor/non-tumor samples. Gene set enrichment analysis (GSEA) results demonstrated that lncRNAs ENST00000520055 and ENST00000535511 shared KEGG pathways with miR-133b target genes.

**Materials and Methods:**

We used microarrays to survey the lncRNA and mRNA expression profiles of colorectal cancer and para-cancer tissues. Gene Ontology (GO) and KEGG pathway enrichment analyses were performed to explore the functions of the significantly dysregulated genes. An innovate method was employed that combined analyses of two microarray data sets to construct a miR-133b-mediated lncRNA-mRNA competing endogenous RNAs (ceRNA) network. Quantitative RT-PCR analysis was used to validate part of this network. GSEA was used to predict the potential functions of these lncRNAs.

**Conclusions:**

This study identifies and validates a new method to investigate the miR-133b-mediated lncRNA-mRNA ceRNA network and lays the foundation for future investigation into the role of lncRNAs in colorectal cancer.

## INTRODUCTION

Human colorectal cancer (CRC) is the third most common cancer in the world, with more than one million new cases each year [1]. The pathogenesis of CRC involves multiple factors, including environmental and genetic variables [2, 3]. Recent reports have revealed that the morbidity and mortality associated with CRC have significantly risen in China [4]. Despite extensive investigation into the molecular mechanisms of CRC and advances in its diagnosis and therapy [5, 6], there are still an estimated 191,000 deaths associated with this disease in China each year [7]. Thus, continued research aimed at identifying novel biomarkers and therapeutic targets is imperative.

Long non-coding RNAs (lncRNAs) are defined as non-coding RNAs of greater than 200 base pairs [8]. Once viewed as transcriptional “noise” without biological functions [9], emerging studies have revealed that lncRNAs play key roles in regulating biological processes, such as genetic imprinting, cell differentiation, the immune response, the cell cycle and apoptosis, as well as human diseases, including various types of cancer [10–12]. Several reports have demonstrated that lncRNAs may function as oncogenes or tumor suppressors in CRC [13–15]. For instance, colorectal cancer-associated lncRNA (CCAL) promotes CRC progression by activating the Wnt/β-catenin signaling pathway through suppression of activator protein 2α [16]. Additionally, LINC01133 inhibits the epithelial-mesenchymal transition and metastasis in CRC by interacting with SRSF6 [17]. However, the specific regulatory mechanisms of lncRNAs in CRC remain largely undefined.

Growing evidence suggests that lncRNAs function as miRNA sponges or competing endogenous RNAs (ceRNAs), reducing the availability of miRNAs for mRNA target binding [18, 19]. Recently, miR-133b acts as an important tumor suppressor in several human cencers [20–22] participates in the migration and invasion of certain types of cancer. miR-133b was found greatly downregulated in CRC and was associated with overall survival and metastasis [23]. Our previous studies validated that miR-133b plays an important role in the progression and metastasis of CRC involving direct targets, such as RhoA, CTGF, and TBPL1 [24–27]. However, whether the ceRNA network is involved in the anti-tumor effect of miR-133b remains unclear. In this study, we identified a miR-133b meditated lncRNA-mRNA ceRNA network by analyzing two microarray data sets in CRC. We then validated a subset of this network using qRT-PCR and predicted the function of two representative lncRNAs using GSEA. These findings illuminate a novel mechanism of CRC pathogenesis and provide new targets for CRC treatment.

## RESULTS

### Expression profile of lncRNAs and mRNAs in CRC

**Figure 1:**
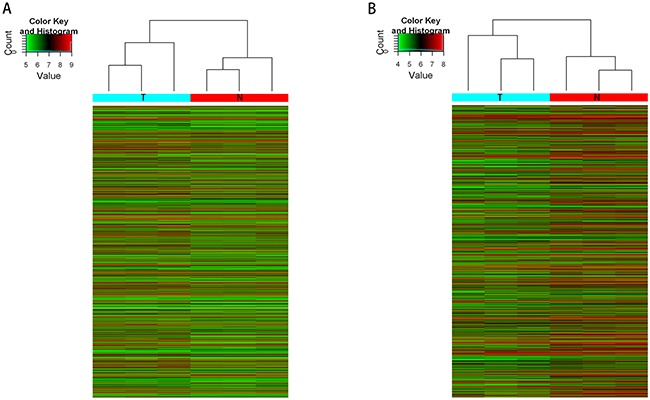
Heat map showing the expression profiles of lncRNAs and mRNAs The maps are based on the expression values of all expressed lncRNAs **A**. and mRNAs **B**. detected by microarray probes. The expression values are represented by a color scale, “red” indicated high relative expression and “green” indicated low relative expression. Each column represents one sample, and each row indicates a transcript. T: tumor tissues. N: normal tissues.

Microarray analysis revealed thousands of lncRNAs and mRNAs expressed in CRC. The expression of 3355 lncRNAs significantly changed in CRC samples relative to matched non-tumor controls. Among these, 763 were upregulated, whereas 2592 were downregulated. A total of 5380 mRNAs significantly changed in expression: 3723 were upregulated, and 1657 were downregulated (fold change≥2.0, P value<0.05). This analysis revealed a different expression pattern of lncRNAs and mRNAs in matched CRC tumor/non-tumor samples (Figure 1). The top 10 upregulated and downregulated lncRNA/mRNAs are listed in Table 1 and Table 2, respectively. The lncRNA/mRNA microarray data have been submitted to NCBI Gene Expression Omnibus and are accessible with the GEO Series accession number GSE90524 (https://www.ncbi.nlm.nih.gov/geo/query/acc.cgi?acc=GSE90524).

**Table 1:** The demographic and biochemical characteristics between cases and controls

Up-regulated lncRNAs		Down-regulated lncRNAs	
Sequence name	Fold-change	Sequence name	Fold-change
ENST00000447469	94.5	TCONS_00006917	248.1
ENST00000515188	40.3	TCONS_00006916	136.8
ENST00000443710	37.2	TCONS_00006918	123.1
TCONS_00007197	34.3	NR_026995	81.1
ENST00000450249	21.3	ENST00000547547	56.2
ENST00000529081	11.5	TCONS_00006919	56.2
NR_029394	11.3	ENST00000458316	35.1
TCONS_00010989	10.0	ENST00000414647	25.7
NR_028308	9.8	ENST00000423329	22.0
NR_033677	8.7	NR_026878	19.4

**Table 2:** The top 10 up-regulated and down-regulated mRNAs

Up-regulated mRNAs		Down-regulated mRNAs	
GeneSymbol	Fold-change	GeneSymbol	Fold-change
IL8	27.7	DRD5	54.2
MMP7	19.2	THEG5	45.0
CXCL5	17.8	GUCA2B	36.7
REG1A	17.1	TMIGD1	35.5
MMP10	15.8	SYPL1	26.7
PPBP	15.6	MRPS18B	25.2
CLEC4E	15.0	NCR2	23.6
KIAA0040	14.9	SHISA8	20.4
ADM	14.8	SORBS2	20.0
MMP3	14.4	GUCA2A	19.1

### Gene ontology (GO) and KEGG pathway analysis

**Figure 2:**
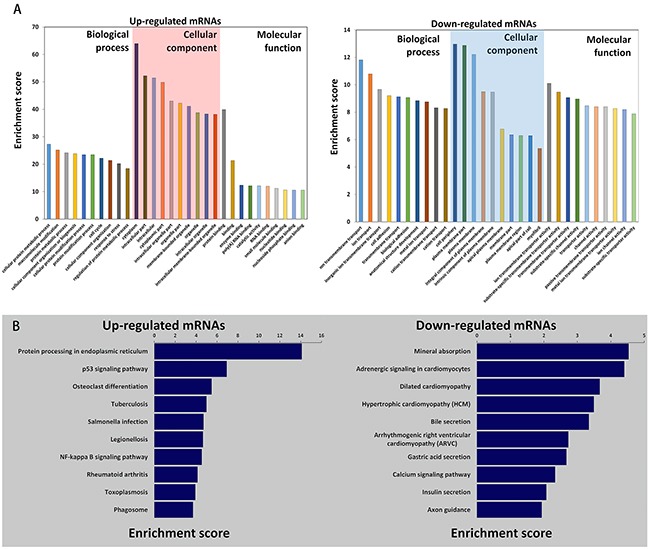
Gene Ontology (GO) and pathway analysis **A**. Go annotation of up- and downregulated mRNAs with the top ten enrichment scores covering domains of biological processes, cellular components and molecular functions. **B**. KEGG pathway enrichment analysis of up- and downregulated mRNAs with the top ten enrichment scores.

Gene ontology (GO) enrichment analysis was performed, revealing the roles of significantly dysregulated mRNAs in CRC. Our data demonstrated that the upregulated mRNAs were genes involved in cellular protein metabolic process, macromolecule modification and protein metabolic process, while the downregulated mRNAs were associated with ion transmembrane transport, ion transport and inorganic ion transmembrane transport (Figure 2A).

KEGG pathway enrichment analysis for significantly dysregulated mRNAs is useful to reveal related pathways and molecular interactions among genes. Our data showed that the upregulated and downregulated mRNAs were each associated with 10 pathways. The p53 signaling pathway was one of the most enriched pathways within the set of upregulated mRNAs (Figure 2B), suggesting that it may be involved in the pathogenesis and development of CRC.

### Combined analysis of the lncRNA/mRNA microarray and the miR-133b overexpression microarrays

**Figure 3:**
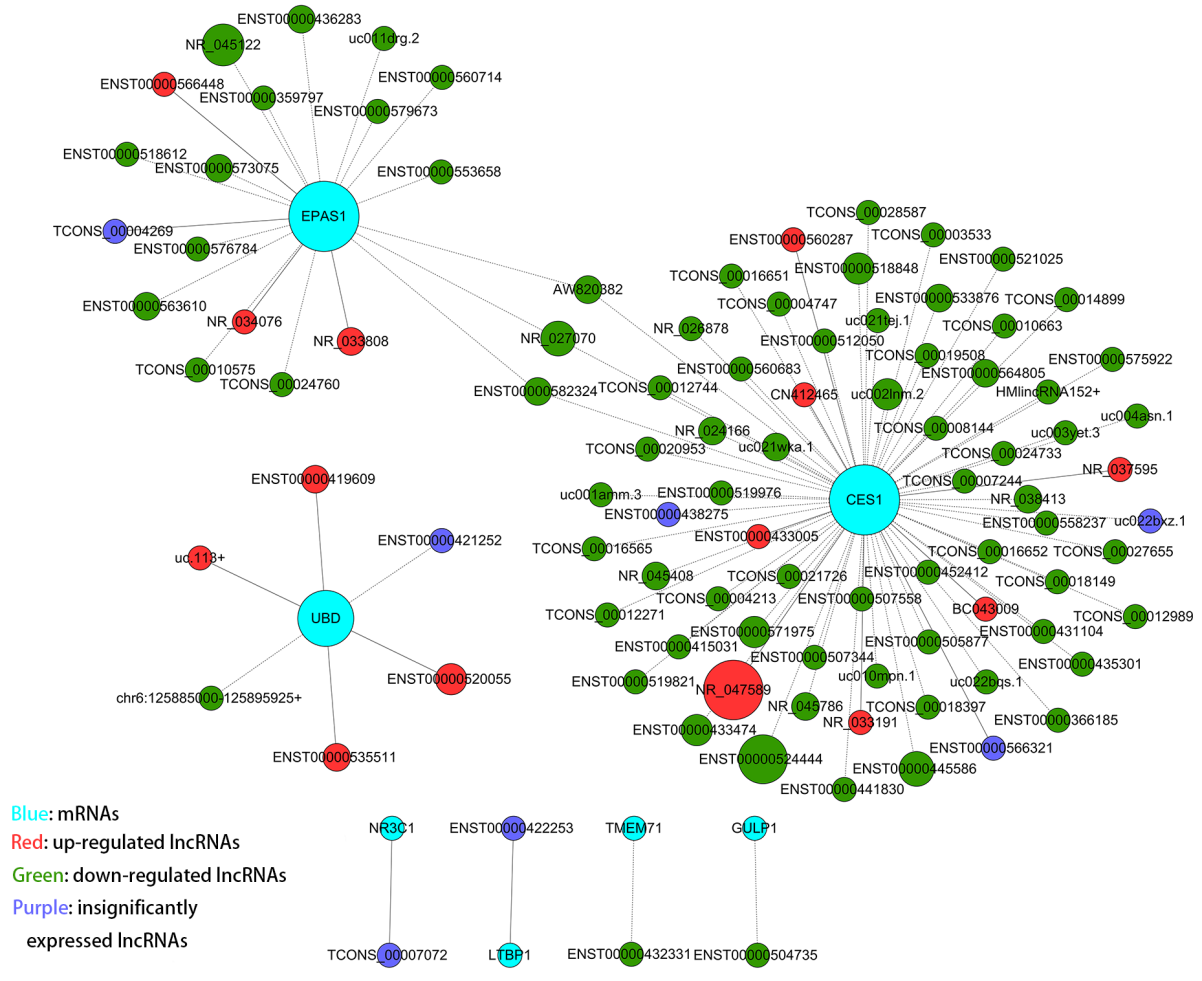
CNC analysis of 8 mRNAs with their associated lncRNAs The network is based on the Pearson correlation coefficient (the absolute value of PCC ≥ 0.995, and FDR <0.05); solid and dashed lines represent positive and negative correlations, respectively).

To determine if miR-133b target genes are regulated by lncRNAs, we performed a combined analysis between our lncRNA/mRNA microarray and a microarray of CRC cell lines that overexpressed miR-133b, which had been reported previously [24]. We obtained 8 genes from the two microarray data sets, including RhoA, TMEM71, LTBP1, EPAS1, UBD, NR3C1, CES1 and GULP1. The inclusion criteria for this analysis were mRNAs downregulated by miR-133b overexpression and upregulated in the lncRNA microarray (fold change>=2.0 and P value <0.05) that also possessed miR-133b miRNA response elements (MREs) (Supplementary Table 1). Using combined coding and non-coding gene (CNC) [28] analysis, we found that 101 lncRNAs significantly correlated with the above genes (Figure 3).

### Construction of an miR-133b-mediated lncRNA-mRNA ceRNA network

**Figure 4:**
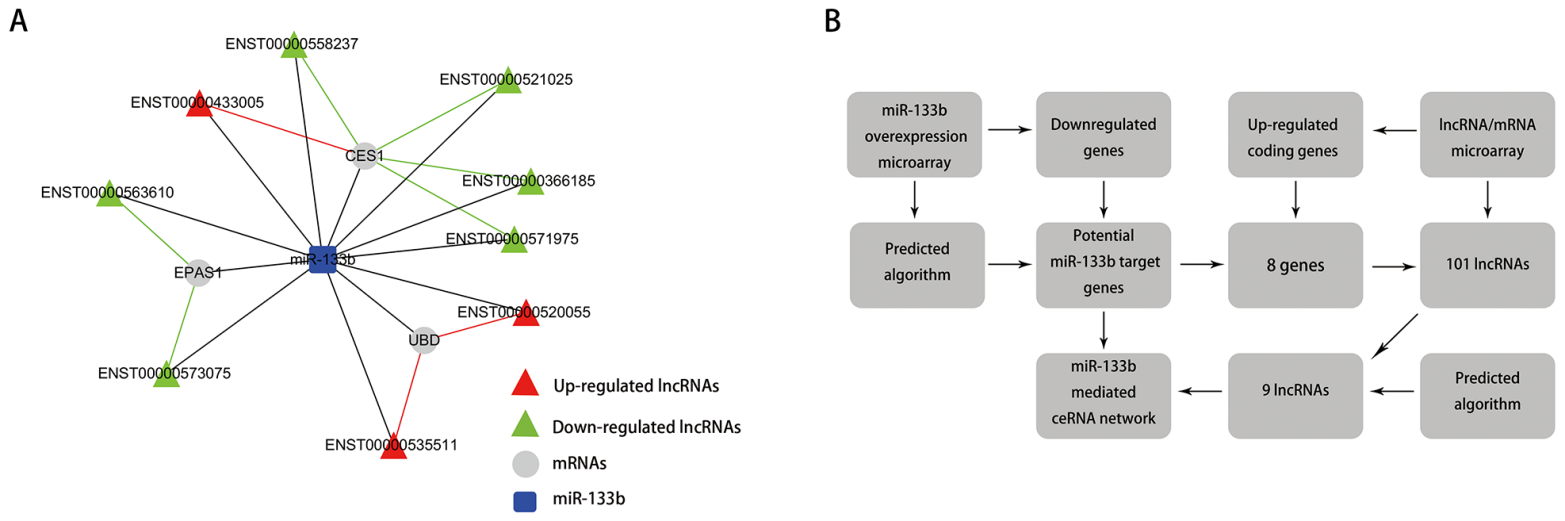
The ceRNA network **A**. miR-133b-mediated lncRNA-mRNA ceRNA network. The competing endogenous RNA network is based on lncRNA/miRNA, lncRNA/mRNA and miRNA/mRNA interactions. In this network, black edges represent sequence matching, red edges represent positive correlation, and green edges represent negative correlation. **B**. the construction of the miR-133b-mediated lncRNA-mRNA ceRNA network.

Since lncRNAs can interact with miRNAs through their MREs within a ceRNA network [18], we searched for putative miR-133b MREs in the above lncRNAs using **RNA22** and **PITA**. A total of 9 lncRNAs were predicted to possess miR-133b binding sites: ENST00000520055, ENST00000535511, ENST00000563610, ENST00000 573075, ENST00000366185, ENST00000433005, ENST 00000521025, ENST00000558237 and ENST000005 71975 (Table 3). Among these, 3 were upregulated and 6 were downregulated in the CRC lncRNA microarray. We then constructed a miR-133b-meditated lncRNA-mRNA ceRNA network, which contains 9 lncRNAs and 3 mRNAs (Figure 4A and Table 4). These RNA interactions may contribute to the progression and metastasis of CRC.

**Table 3:** 9 lncRNAs possess miR-133b MREs

LncRNA	Sequence	algorithms
ENST00000520055	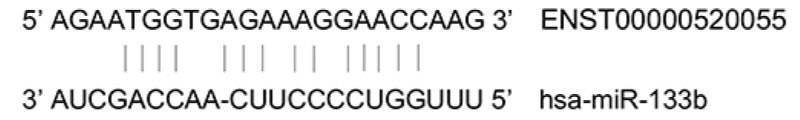	PITA RNA22
ENST00000535511	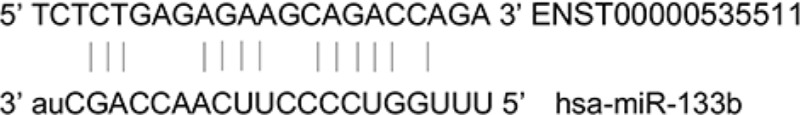	PITA RNA22
ENST00000563610	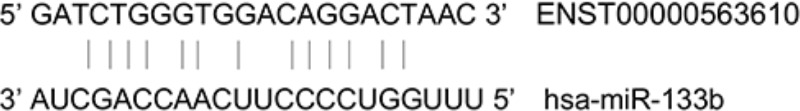	PITA RNA22
ENST00000573075	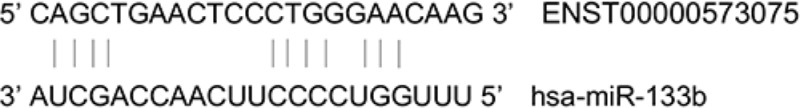	PITA RNA22
ENST00000366185	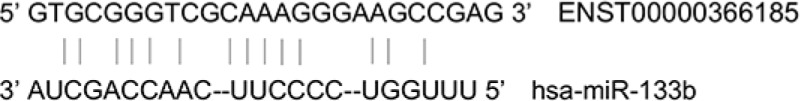	PITA RNA22
ENST00000433005	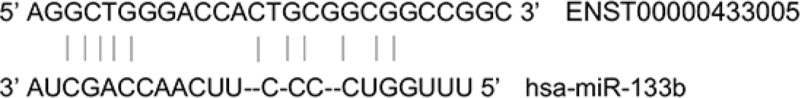	PITA RNA22
ENST00000521025	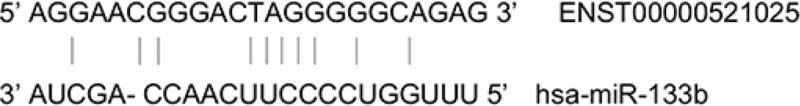	PITA RNA22
ENST00000558237	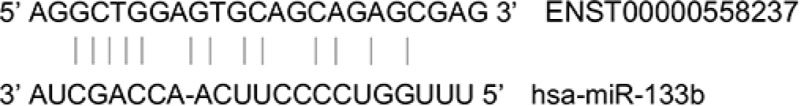	PITA RNA22
ENST00000571975	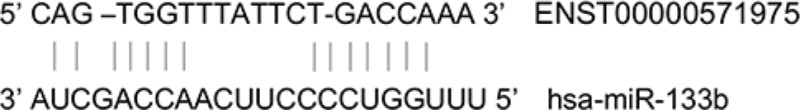	PITA RNA22

**Table 4:** miR-133b mediated lncRNA-mRNA ceRNA network

mRNAs	related lncRNAs
UBD	ENST00000520055, ENST00000535511
EPAS1	ENST00000563610, ENST00000573075
CES1	ENST00000361155, ENST00000433005
	ENST00000521025, ENST00000558237
	ENST00000571975

### qRT-PCR validation of components of the miR-133b meditated lncRNA-mRNA ceRNA network

**Figure 5:**
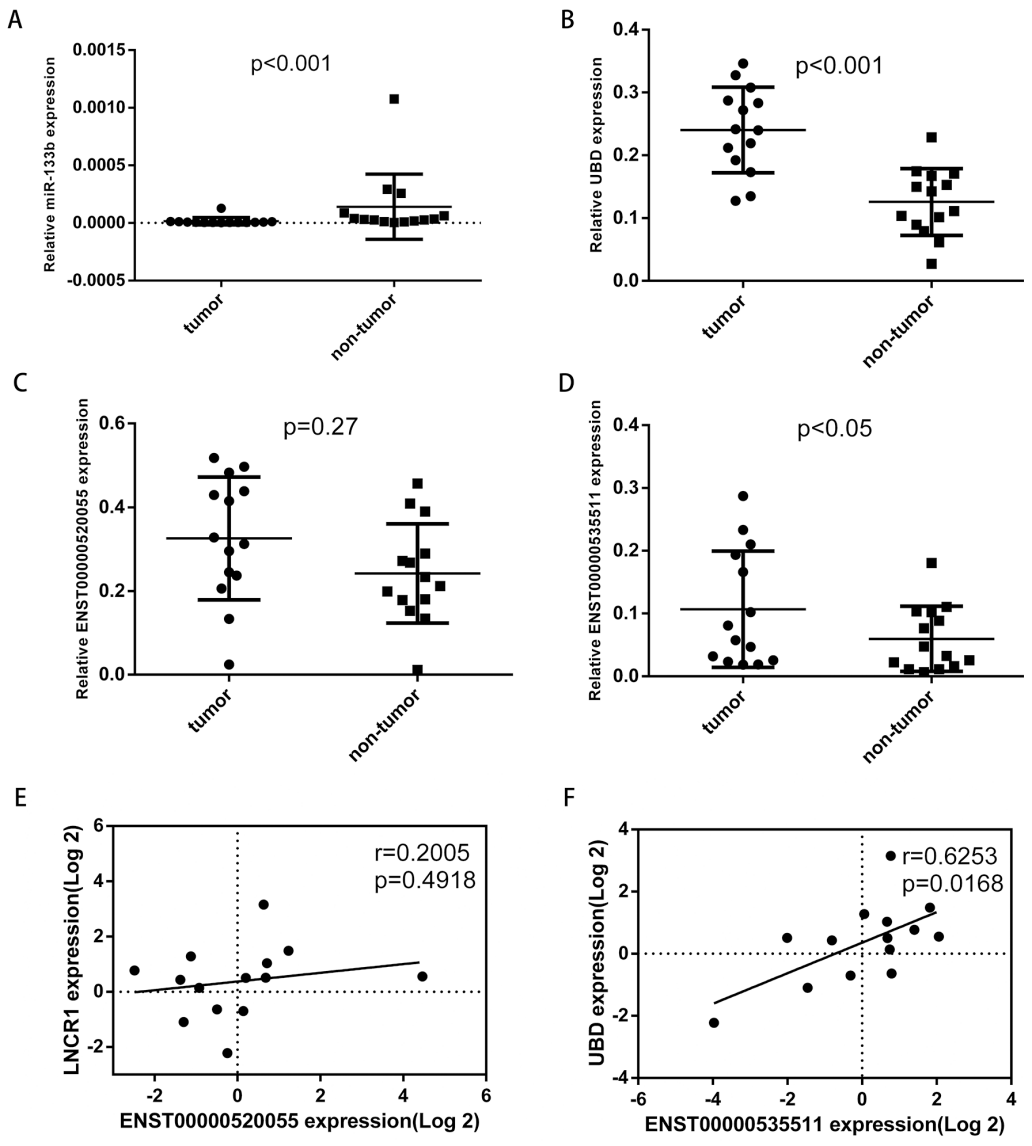
The expression levels of miR-133b, UBD, ENST00000520055, ENST00000535511 in 14 pairs of CRC tumor and non-tumor tissues, and the relationship between UBD, ENST00000520055, and ENST00000535511 Relative expression levels of miR-133b **A**. UBD **B**. ENST00000520055 **C**. ENST00000535511 **D**. **E**. The relationship between ENST00000520055 and UBD expression levels. **F**. The relationship between ENST00000535511 and UBD expression levels.

UBD has been reported to be overexpressed in colon cancer and may contribute to the progression of colon carcinogenesis, as well as function as a prognostic indicator [29, 30]. Thus, we chose miR-133b, UBD and related lncRNAs to validate the potential ceRNA network. qRT-PCR was performed to assess their expression levels in 14 pairs of matched colorectal tumor/non-tumor sample. This analysis revealed that miR-133b was significantly downregulated in CRC tumor samples (Figure 5A), while UBD and lncRNA ENST00000535511 (Figure 5B and Figure 5D) were significantly upregulated in CRC tumor samples. The expression of lncRNA ENST00000520055 (Figure 5C) was not significantly upregulated (P=0.27) in CRC tumor samples, potentially due to the insufficient sample size. A significantly positive correlation between UBD and lncRNA ENST00000535511 was also observed (Figure 5F). These co-expression characters were in accordance with ceRNA hypothesis.

### Gene set enrichment analysis of coding genes that correlated with miR-133b targeted lncRNAs

**Figure 6:**
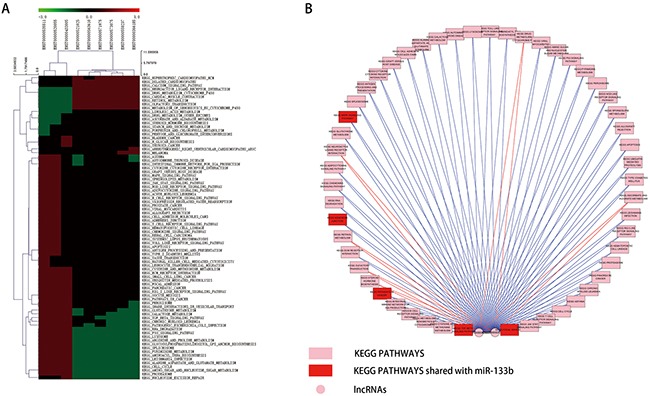
GSEA results of the 9 lncRNAs **A**. heatmap of KEGG pathway signatures correlated with the 9 lncRNAs. **B**. Visualization of the GSEA results of ENST00000520055 and ENST00000535511 based on Cytoscape. The red nodes represent the KEGG PATHWAYS shared with miR-133b predicted target genes.

To evaluate the potential roles of lncRNAs in tumorigenesis-related biological processes, we conducted gene set enrichment analysis (GSEA) on the coding genes whose expression correlated with these lncRNAs [31]. Significantly enriched gene sets (|NES| > 1, FDR< 0.2) are visualized as a heatmap in Figure 6A. This analysis also revealed that ENST00000520055 and ENST00000535511 positively correlated with several KEGG pathways: TGF BETA SIGNALING PATHWAY, PATHWAYS IN CANCER, MAPK SIGNALING PATHWAY, ADHERENS JUNCTION and FOCAL ADHESION (Figure 6B). This result is consistent with the KEGG pathways of predicted miR-133b target genes [32].

## DISCUSSION

The strength of the ceRNA hypothesis is its potential to account for the function of a substantial proportion of uncharacterized lncRNAs [33], and several recent reports provide support for this hypothesis [34–36]. While it has been argued that the expression levels of individual lncRNAs are not sufficient to sequester miRNAs and inhibit their function [37–39], ceRNA concentration may be spatially or temporally enriched or exhibit enhanced stability to meet the high expression requirement for ceRNA activity [40–42]. Furthermore, miRNAs expressed at low levels may be more susceptible to ceRNA regulation [39]. Our previous studies demonstrated that miR-133b was downregulated in primary CRC, and even more so in liver metastasis [43]; therefore, identifying the ceRNAs of miR-133b is essential. Herein, we provided a new method for the elucidating specific miRNA related ceRNA networks.

This report describes a novel method for constructing specific miRNA-mediated lncRNA-mRNA ceRNA networks. The most widely used methods for exploring potential lncRNA-mRNA ceRNA networks largely depend on shared MREs, predicted by miRNA target discovery algorithms [44–46]. However, we first identified miR-133b-target gene pairs based on microarray analysis of CRC cell lines overexpressing miR-133b and then obtained lncRNAs that correlated with them by combining the analysis with a lncRNA microarray. We then incorporated a bioinformatic prediction tool to construct a miR-133b-mediated lncRNA-mRNA ceRNA network. To our knowledge, this is the first report that constructs a miRNA-mediated lncRNA-mRNA ceRNA network using combined analysis of miRNA overexpression and lncRNA microarrays. Distinct from other methods, we searched for the lncRNAs involved in the ceRNA network based on the putative miR-133b-target gene pairs. One of these pairs was validated [26]. Thus, we could identify miR-133b-regulated mRNAs as well as lncRNAs from this analysis. The benefit of our method was that we could acquire key lncRNAs and miRNA-target gene pairs; this hypothesis was supported by GSEA. These results identifying a novel ceRNA network are more reliable because they are based on two microarrays.

Our method also had some limitations. First, we could not identify a large miRNA-mediated lncRNA-mRNA ceRNA network by this method. Additionally, the miR-133b-mediated lncRNA-mRNA networks that were constructed require further investigation. Although we validated some of the components of this network in CRC, more CRC tumor and paired non-tumor samples need to be analyzed. Finally, since this approach is based on changes in transcript level, targets regulated by translational repression will not be identified. Our future work will focus on further validating the miR-133b-mediated lncRNA-mRNA ceRNA network and elucidating the role of this network in the progression and metastasis of CRC.

In conclusion, this study identifies and validates a new method to investigate the miR-133b-mediated lncRNA-mRNA ceRNA network and provides a novel approach to identify specific miRNA targets and related lncRNAs.

## MATERIALS AND METHODS

### Tissue samples

Surgical samples of CRC tissues and their adjacent noncancerous tissues were obtained from The Third Xiangya Hospital of Central South University from March 2014. The adjacent noncancerous tissues were 5 cm from the edge of tumor; this tissue contained no tumor cells, based on an evaluation by an experienced pathologist. There was no radiotherapy or chemotherapy prior to the operation. The Human Research Ethics Committee from Central South University approved the entire study, and written informed consent was obtained from all subjects.

### RNA extraction

Samples were immediately frozen in liquid nitrogen after surgical dissection. Total RNA was extracted using TRIzol Reagent (Invitrogen, CA, USA) according to the manufacturer's protocol. RNA quantity was measured by the NanoDrop ND-2000 spectrophotometer (OD 260 nm, NanoDrop, Wilmington, DE, USA), and RNA integrity was assessed using standard denaturing agarose gel electrophoresis.

### LncRNA and mRNA microarrays

Human lncRNA Microarray V3.0 was manufactured by Arraystar Inc (MD, USA) and covered more than 40,000 lncRNAs and 20,000 mRNAs in the human genome. The microarray hybridization, collection of expression data and analysis of microarray data were performed by KangChen Bio-tech, Shanghai, China.

### Gene ontology analysis and KEGG pathway analysis

Gene ontology (GO) analysis provides a controlled vocabulary to describe gene and gene product attributes in any organism (http://www.geneontology.org). This ontology covers three domains: biological processes, cellular components and molecular functions. Fisher's exact test was used to the detect overlap between the differentially expressed genes and the GO annotation list beyond that which would be expected by chance. The P value denotes the significance of GO term enrichment among differentially expressed genes (P value ≤ 0.05 is recommended).

Pathway analysis is used to map genes to KEGG pathways. The P value (EASE-score, Fisher P value or hypergeometric P value) denotes the significance of the pathway correlations (P value ≤ 0.05 is recommended). The GO and KEGG pathways analyses were performed by KangChen Bio-tech, Shanghai, China.

### qRT-PCR analysis

Total RNA was extracted from CRC tumor/non-tumor samples using TRIzol reagent (Invitrogen). It was then converted into cDNA using the TOYOBO RT kit according to the manufacturer's instructions. PCR was performed in a total reaction volume of 20 μL containing 10 μL RNA-direct™ SYBR® Green Realtime PCR Master Mix (2×) (TOYOBO), 0.8 μL forward primer (10 μM), 0.8 μL reverse primer (10 μM), 0.6 μL cDNA, and 7.8 μL double-distilled water. The primers used in this study are summarized in Supplementary Table 2. All reactions were performed using the Roche LightCycler 480 thermal cycler (Roche, Switzerland). The amplification conditions were as follows, 2 min at 98 °C; 40 cycles of 10 s at 98 °C, 10 s at 60 °C, and 30 s at 68 °C; and a final extension for 5 min at 72 °C. Amplification efficiency was evaluated via standard curve analysis. All lncRNA and mRNA expression data were normalized to GAPDH, and miR-133b was normalized to U6. Each experiment was repeated three times. The relative expression of the genes were calculated using the 2^−ΔCT^ method.

### CNC analysis

The CNC analysis was based on calculating the Pearson correlation coefficient (PCC) between the expression levels of coding and noncoding genes. We subsequently screened based on the Pearson correlation coefficient using the selection parameters PCC ≥ 0.995 and FDR<0.05. The co-expression network was illustrated using Cytoscape (v3.4.0). Analyses were performed by KangChen Bio-tech, Shanghai, China.

### Construction of the miR-133b-related ceRNA network

Two criteria were applied in the selection of genes to include in the construction of the miR-133b-mediated ceRNA network (Figure 4B): (i) lncRNA screen: lncRNAs that were dysregulated based on a fold change of  ≥2.0 and P value<0.05 that significantly correlated with miR-133b predicted target genes; and (ii) lncRNAs that possessed miR-133b MREs, as predicted by RNA22 (https://cm.jefferson.edu/rna22/Precomputed/) and PITA (http://genie.weizmann.ac.il/pubs/mir07/mir07_data.html).

### GSEA of lncRNA

GSEA was performed by the JAVA program (http://www.broadinstitute.org/gsea) using MSigDB C2 CP and the canonical pathways gene set collection (1320 gene sets available). A total of 1000 random sample permutations were carried out, and the meaningful threshold was set at |NES| > 1, FDR< 0.2[31]. Cytoscape was used for visualization of the GSEA results. Analyses were performed by KangChen Bio-tech, Shanghai, China.

### Statistical analysis

All statistical analyses were performed using SPSS 17.0 software (SPSS Inc.) unless otherwise noted. Differences in the expression levels of miR-133b, UBD and lncRNAs between CRC tumor samples and paired non-tumor samples were evaluated using a two-tailed t-test. Differences were considered to be statistically significant at P<0.05. The microarray data was analysed using unpaired t-test.

## SUPPLEMENTARY MATERIALS FIGURES AND TABLES




