# Supplementary material for: Comprehensive analysis of differentially expressed profiles of lncRNAs and construction of miR-133b mediated ceRNA network in colorectal cancer
Source: Oncotarget. 2017 Feb 3;8(13):21095–105. doi: 10.18632/oncotarget.15045 (PMC5400568; doi:10.18632/oncotarget.15045)
Supplement: Supplementary file 1 [file oncotarget-08-21095-s001.pdf]

Comprehensive analysis of differentially expressed profiles of lncRNAs and construction of miR-133b mediated ceRNA network in colorectal cancer

SUPPLEMENTARY TABLES

Supplementary Table 1: 8 mRNAs possess miR-133b MREs.

See Supplementary File 1.

Supplementary Table 2: Primer sequence list

Gene	Primer sequence
miR-133b	Forward: 5'-GCAGGTTTGGTCCCCTTCAAC-3'
	Reverse: 5'-GTGCAGGGTCCGAGGT-3'
U6	Forward: 5'-CTCGCTTCGGCAGCACA-3'
	Reverse: 5'-AACGCTTCACGAATTTGCGT-3'
ENST00000520055	Forward: 5'-GGCCTCAGACTGTTTCTCGT-3'
	Reverse: 5'-CCCTCATCTCCCTTTAGCCC-3'
ENST00000535511	Forward: 5'-TAGAACATCTCCAGCCATCAT-3'
	Reverse: 5'-GTTAAGGTGAACTCTGAAGGT-3'
UBD	Forward: 5'-CCGTTCCGAGGAATGGGATTT-3'
	Reverse: 5'-GCCATAAGATGAGAGGCTTCTCC-3'
GAPDH	Forward: 5'-TGCACCACCAACTGCTTAGC-3'
	Reverse: 5`- GGCATGGACTGTGGTCATGAG-3`
