# Supplementary material for: Comprehensive analysis of differentially expressed profiles of lncRNAs and construction of miR-133b mediated ceRNA network in colorectal cancer
Source: Oncotarget. 2017 Feb 3;8(13):21095–105. doi: 10.18632/oncotarget.15045 (PMC5400568; doi:10.18632/oncotarget.15045)
Supplement: Supplementary file 2 [file oncotarget-08-21095-s002.docx]

Supplementary Table 1：8 mRNAs possess miR-133b MREs

| **mRNAs** | **Sequence** | **algorithms** |
| --- | --- | --- |
| RhoA | 5’ CAGTTT-TTTGAAAATGGGCTCAAC 3’ RhoA  3’ AUCGACCAAC--UUCCCCUGGUUU 5’ hsa-miR-133b | Tarbase |
| TMEM71 | 5’ CAACTGGTTGAAGGGGACCAGG 3’ TMEM71  3’ auCGACCAACUUCCCCUGGUUU 5’ hsa-miR-133b | RNA22 |
| LTBP1 | 5’ CCGAUAGGUGUGGCAGACCAAAU 3’ LTBP1  3’ AUCGACCAACUUCCCCUGGUUU 5’ hsa-miR-133b | Targetscan  RNA22 |
| EPAS1 | 5’ AAGCTAA AGGAGGAGCCCGAG 3’ EPAS1  3’ AUCGACCAACUUCCCCUGGUUU 5’ hsa-miR-133b | RNA22 |
| UBD | 5’ GGGGTGTTGGCAGGGGTCAAA 3’ UBD  3’ AUCGACCAACUUCCCCUGGUUU 5’ hsa-miR-133b | RNA22 |
| NR3C1 | 5’ TAGCCCAGGAAAGGGG-CCAGC 3’ NRC1  3’ AUCGACCAACUUCCCCUGGUUU 5’ hsa-miR-133b | RNA22 |
| CES1 | 5’ TGGCTGCCCTGCGCTGGGTCCAGG 3’ CES1  3’ AUCGACCA ACUU-CCCCUGGUUU 5’ hsa-miR-133b | RNA22 |
| GULP1 | 5’ CGCGAAGAGGGAGGGGACCGAA 3’ GULP1  3’ AUCGA CCAACUUCCCCUGGUUU 5’ hsa-miR-133b | RNA22 |
